# Validation of Fixed Ultrasonography for Achilles Tendon Assessment: A Reliability Study

**DOI:** 10.3390/diagnostics14192221

**Published:** 2024-10-05

**Authors:** Raquel Alabau-Dasi, Gabriel Dominguez-Maldonado, Ana Belen Ortega-Avila, Luis M. Gordillo-Fernandez, Mercedes Ortiz-Romero, Juan Manuel Melchor-Rodriguez, Gabriel Gijon-Nogueron

**Affiliations:** 1 Facultad de Ciencias de la Salud, Departamento de Enfermería y Podología, Universidad de Malaga, 29071 Malaga, Spain; rada@uma.es (R.A.-D.); gagijon@uma.es (G.G.-N.); 2 Programa de Doctorado, Facultad de Ciencias de la Salud, Universidad de Malaga, 29071 Malaga, Spain; gdominguez@us.es; 3 Departamento de Podología, Universidad de Sevilla, 41092 Sevilla, Spain; lgordillo@us.es (L.M.G.-F.); mortiz17@us.es (M.O.-R.); 4 Instituto de Investigación Biomédica de Málaga y Plataforma en Nanomedicina-IBIMA Platform BIONAND, 29590 Malaga, Spain; 5 Biostatistics Teaching Unit, Department of Statistics and Operations Research, Faculty of Medicine, University of Granada, 18016 Granada, Spain; jmelchor@go.ugr.es; 6 Biomedical Research Institute—ibs.GRANADA, 18012 Granada, Spain

**Keywords:** Achilles, reliability, tendon, ultrasonography, validation

## Abstract

**Background:** It is important to highlight the advantages of ultrasound in assessing muscular and tendinous behavior due to its non-invasive nature and capacity for dynamic studies. However, evaluating tendons via ultrasound can be challenging given the complexity of anisotropic phenomena related to collagen fiber arrangement. This study aims to validate the reliability of fixed ultrasound compared to manual acquisition in measuring Achilles tendon thickness. **Method:** Twenty participants, six men and fourteen women, were recruited. Ultrasound was used to measure the Achilles tendon’s thickness at two specific points (4 and 6 cm from the calcaneal insertion of the Achilles tendon). The measurements were conducted by two examiners, one with previous experience and another without. **Results:** The measurements at 6 cm from the calcaneal insertion showed α = 0.996, α = 0.998 for measurements at 4 cm using manual acquisition, and α = 0.997 for measurements with fixed ultrasound at rest. For the weight-bearing and ankle dorsiflexion measurements, the reliability was excellent (α = 0.999 and α = 1.000). **Conclusions:** The findings demonstrated excellent reliability in the ultrasound measurements of the Achilles tendon’s thickness, even when performed by different evaluators and under load-bearing conditions. This study suggests the clinical utility of assessing anatomical structures under load, enhancing ultrasound’s applicability beyond the examination table. It is concluded that fixed ultrasound acquisition exhibits excellent reliability in measuring the Achilles tendon’s thickness, offering potential benefits for precise diagnosis of pathologies, planning surgical interventions, and reducing possible errors related to operator variability.

## 1. Introduction

In the last decade, ultrasound has become an important complementary tool to physical examination and other diagnostic techniques in the clinical management of musculoskeletal pathologies [1]. The Achilles tendon (AT) is a load-bearing tendon crucial for the dorsiflexion of the tibiofibular joint, playing a fundamental role in the biomechanics of the foot and ankle. The proper assessment of this structure is vital in clinical settings for diagnosing and treating various foot and ankle pathologies [2].

Ultrasound presents unique advantages as a non-invasive diagnostic technique that allows the dynamic observation of muscular and tendinous behavior. This is particularly important for the AT, as ultrasound enables the visualization of the tendon’s internal structure during movement, providing detailed information that is not accessible through other static imaging techniques [1,3].

However, evaluating the AT can be challenging due to anisotropic phenomena associated with the collagen fibril arrangement within the tendon [4,5]. Traditionally, these evaluations are performed with the patient at rest, which can be a disadvantage since the AT is a load-bearing tendon. Assessing the AT under load-bearing conditions (with the patient standing) can provide more relevant information that aligns with the tendon’s functional role in daily activities [6].

In ultrasound examinations of the AT, parameters such as tendon thickness, cross-sectional area, orientation of muscle fibers at tendon insertion, and echogenicity are assessed [7]. Typically, clinicians conduct these examinations with the patient lying prone on the examination table, with feet hanging off the edge and the ankle slightly dorsiflexed [8,9]. Evaluating the resting Achilles tendon may lead to the loss of crucial information regarding its functionality under load [10,11]. In contrast, performing ultrasound in a weight-bearing posture facilitates the monitoring of the tendon function during static and dynamic loading, resulting in diagnoses that more closely reflect the structure’s operational characteristics [12].

Ultrasound evaluations of the AT are commonly used in clinical settings to differentiate between tendons with and without tendinopathy [13,14]. Despite the apparent simplicity of visualizing the AT via ultrasound, intra- and inter-rater variability can arise from the examiner’s experience and skill, potentially affecting the results [15]. Measurement errors can also stem from the protocol, equipment used, and biological variability among subjects [16]. Common sources of variability include probe pressure on the skin and the angle at which the tissue is imaged, which can influence measurements [17].

Given the substantial operator dependence in ultrasound measurements, there is an urgent need to establish standardized protocols for ultrasound measurement [18,19]. The aim of this study is to validate the reliability of fixed ultrasound compared to manual acquisition by measuring the thickness of the AT under both non-weight-bearing and weight-bearing conditions. We hypothesize that fixed ultrasound under load-bearing conditions can provide reliable and consistent measurements, offering a valuable tool for clinical practice. This study aims to validate the reliability of fixed ultrasound in load-bearing versus manual acquisition by measuring the thickness of the AT in non-weight-bearing (patient at rest on the examination table) and weight-bearing (patient in a standing position performing maximum ankle dorsiflexion [20]) conditions.

## 2. Materials and Methods

### 2.1. Design

This study was an intra-rater reliability and inter-rater concordance study.

### 2.2. Population

The participants were students recruited from the University of Malaga from March to May 2023. Inclusion criteria were individuals over 18 years old, without previous Achilles tendon pathology, and capable of walking autonomously. Exclusion criteria included disorders of the lower extremities, systemic, vascular, and/or joint diseases affecting the musculoskeletal system (such as diabetes, rheumatoid arthritis, collagen disorders), pregnancy, prior surgeries involving the lower extremities, and the use of orthopedic or non-orthopedic treatments on the lower extremities at the time of data collection. Although this sample was composed of university students, the inclusion and exclusion criteria aimed to ensure that the participants represented a healthy, general population without specific Achilles tendon pathologies. The sample size calculation was performed prior to recruitment, ensuring that the study was adequately powered to detect a significant correlation between measurements.

### 2.3. Procedure

The subjects who were evaluated by the team member in face-to-face interviews, where the procedure was explained, the informed consent form was signed, and a demographic history form (including age, height, weight, sex, smoking habits, current injury status, allergies, medications, previous surgeries, type of sport, and number of weekly workouts) was completed. This information allowed the establishment of the sample in accordance with the inclusion and exclusion criteria.

The ultrasound examination of the participants was conducted by an experienced sonographer with 5 years of experience (RA) (Examiner 1) and an evaluator without previous experience (AV) (Examiner 2), who had undergone training in musculoskeletal ultrasound examination of the foot and ankle. Both examiners received instructions and training on the test setup before the study began to ensure they followed the study protocol. The training, conducted for both operators, served as a refresher on the theoretical framework, including the physical foundations of the exams and the methods of palpation and measurement. To ensure blinding between the two examiners, the measurements from each examiner were kept confidential and were not shared between them at any point during the study. Furthermore, the examiners performed the assessments independently and in separate rooms, minimizing any potential bias or influence on the other’s results. Subsequently, the sonographers analyzed the Achilles tendon (AT) following protocols defined in previous studies, which included patient and probe positioning, and the identification of tendon edges in the resulting images [21,22].

### 2.4. Protocol Scanning

The ultrasound images were collected using a portable musculoskeletal ultrasound system, the MyLab™ Sigma Elite (Esaote, Italy), equipped with a high-density linear probe (4–15 MHz). The depth of the image field was set at 3.5 cm, the gain was set at 85 dB, the probe frequency was set at 14 MHz, and a single focal zone (set at a depth of 0.5 cm) was placed at the level of the AT. To fix the probe to the ankle a custom-designed device was created to ensure consistent positioning of the ultrasound probe throughout the evaluation process. The device is adjustable to accommodate different ankle sizes and utilizes a secure strap mechanism to maintain constant pressure on the probe, preventing movement during the examination. The material used for the device is lightweight, hypoallergenic, and designed to minimize any interference with the ultrasound signal. A device was designed to keep the probe fixed at all times. This device is in the process of being patented by the Spanish Patent and Trademark Office (OEPM). Each examiner independently conducted the scanning in a random order within the same session to assess inter-examiner reliability. Additionally, the expert examiner repeated the measurements one week later to evaluate intra-subject reliability. Researchers were blinded to any prior measurements during the scanning sessions.

To assess the AT thickness, images were captured at rest with probe fixation and manual acquisition. Subsequently, the same procedure was performed with the participant standing in a maximum ankle dorsiflexion position.

Examiner 1 initially performed a measurement at rest using manual acquisition, with the patient lying prone and their feet hanging off the edge of the examination table in a relaxed position [23]. To carry out the measurements, the distance between the most proximal point of the posterior edge of the calcaneus and the distal end of the musculotendinous junction of the soleus was determined. This process involved identifying anatomical reference points, marking them on the skin to ensure consistency, and capturing images of the tendon to calculate its thickness. The procedure was repeated for both legs.

**Figure 1:**
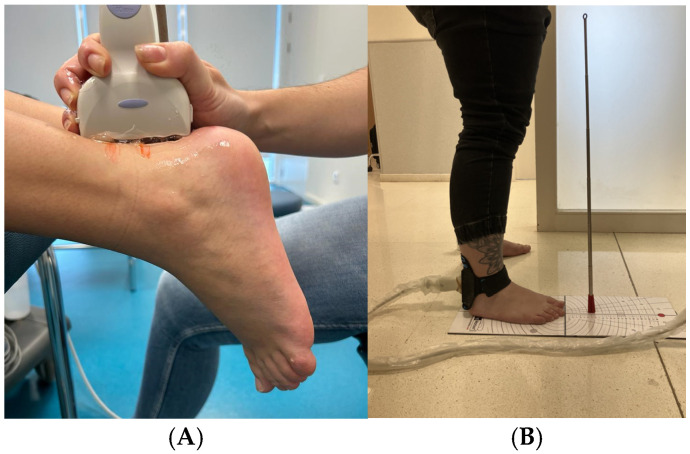
Patient positioning during the ultrasound procedure for Achilles tendon evaluation. (**A**) The patient is lying prone with their feet hanging off the edge of the examination table. (**B**) The patient is standing in a static load-bearing position on the floor. Anatomical reference points marked on the skin and the placement of the ultrasound probe over the tendon area are shown.

Subsequently, the same Examiner 1 repeated the same process using fixed ultrasonography. In this phase, the previously marked anatomical reference points were used to ensure the accuracy of the measurements. Additionally, the procedure was performed with the patient in a weight-bearing position, standing directly on the floor (Figure 1A,B). This posture was deliberately chosen to replicate the typical functional conditions under which the Achilles tendon bears weight, ensuring that the measurements obtained more accurately reflect its behavior under real tension [21,23,24].

After a 5 min rest for the subject, Examiner 2, in a separate adjacent room, replicated the same measurements as Examiner 1 using fixed ultrasonography.

Measurements of the AT thickness were obtained at 4 cm and 6 cm distances from the insertion of the AT to the calcaneus. These points had been previously marked on the skin [13,19,23]. Following each scan, the skin marks were removed to ensure blinding of the results among researchers [23,25,26]. All sonograms were stored in the ultrasound machine (MyLab™ Sigma Elite, Esaote, Italy).

### 2.5. Statistical Analysis

**Figure 2:**
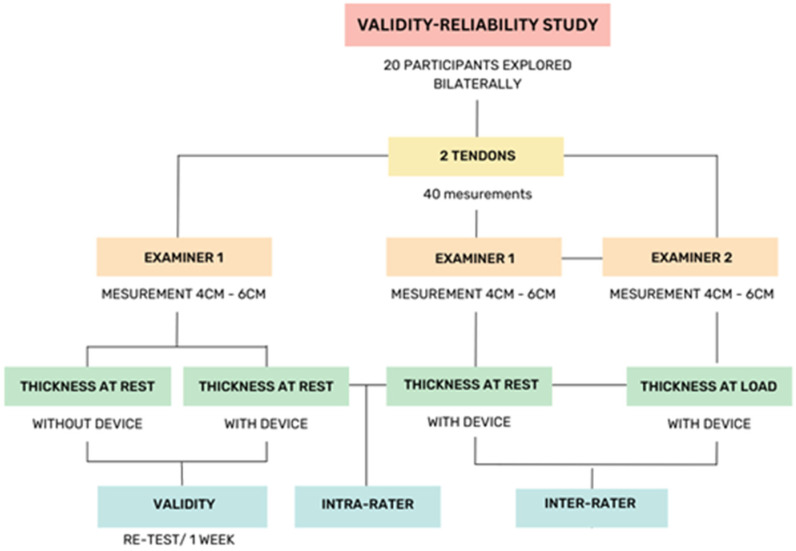
Sequence of the intra-rater reliability procedure.

It was determined that a sample of 20 participants was sufficient based on a sample size calculation required to detect a correlation coefficient significantly different from zero (r = 0.7) with a 95% confidence interval and a statistical power of 80%. The correlation coefficient of 0.7 was chosen based on previous research in musculoskeletal ultrasound studies, which has reported similar values for assessing the reliability and validity of ultrasound measurements. Specifically, studies assessing tendon measurements have demonstrated correlation coefficients within this range as indicative of strong associations. Furthermore, previous studies in the field of Achilles tendon ultrasound reliability, such as [9], have consistently used sample sizes between 15 and 25 participants, showing that these sample sizes provide sufficient power to detect meaningful relationships and ensure reliable measurements [23,26,27] (Figure 2).

The statistical analyses were performed using Jamovi Project version 2.3, 2022. Mean and standard deviation were used for descriptive statistics with 95% confidence intervals (CI). The data were normally distributed, as confirmed by a visual inspection of QQ plots, kurtosis and skewness coefficients, and the Kolmogorov–Smirnov test [19,21]. To determine the reliability of measurements between evaluators, Intraclass Correlation Coefficients (ICCs) were calculated using a two-way random effects model and an absolute agreement type [26]. The following criteria were used to assess reliability coefficients: very poor (<0.20), poor (0.21–0.40), moderate (0.41–0.60), good (0.61–0.80), and excellent (0.81–1.00) [26,28].

Additionally, in a Bland–Altman plot, 95% limits of agreement (LOA) were calculated. Bland–Altman plots were constructed to assess the presence of systematic errors. The difference between each pair of measurements (Y-axis) is plotted against the mean of both measurements (X-axis), with the overall mean difference and the 95% agreement limits completing the graphical information. LOA is presented as the difference between the mean difference and the upper and lower LOA to comprehend the result in a clinical context. Passing–Bablok regression analysis was applied to evaluate potential systematic bias in the measurement.

## 3. Results

### 3.1. Sample Description

The sample consisted of fourteen women and six men, with a mean age of 22.55 ± 2.32 years and a mean BMI of 23.61 ± 2.97 (Table 1).

### 3.2. Ultrasound Data

The ultrasound data for the thickness of the Achilles tendon obtained by the two examiners are shown in Table 2. The mean comparisons between Examiner 1, who was more experienced, and Examiner 2 at rest were 4.58 mm and 4.66 mm for the thickness at 4 cm from the calcaneal insertion and 4.67 mm and 4.66 mm at 6 cm from the insertion, respectively.

### 3.3. Reliability

The ICC for all measurements taken at 6 cm from the insertion was 0.909 (95% CI = 0.861 to 0.945). The ICC was 0.926 (95% CI = 0.882 to 0.957) for measurements at 4 cm from the insertion. For weight-bearing measurements, an ICC of 0.876 (95% CI = 0.802 to 0.928) was obtained at 6 cm from the insertion and an ICC of 0.894 (95% CI = 0.826 to 0.940) was obtained at 4 cm from the insertion (Table 2).

### 3.4. Inter-Rater Reliability

Regarding inter-rater reliability, Cronbach’s alpha coefficient was used, resulting in α = 0.996 for measurements at 6 cm from the calcaneal insertion and α = 0.998 for measurements at 4 cm using manual acquisition. For measurements with fixed ultrasound at rest, α = 0.997, and for weight-bearing measurements with ankle dorsiflexion, these measurements were excellent (α = 0.999 and α = 1.000) (Table 2).

### 3.5. Bland–Altman Plots

**Figure 3:**
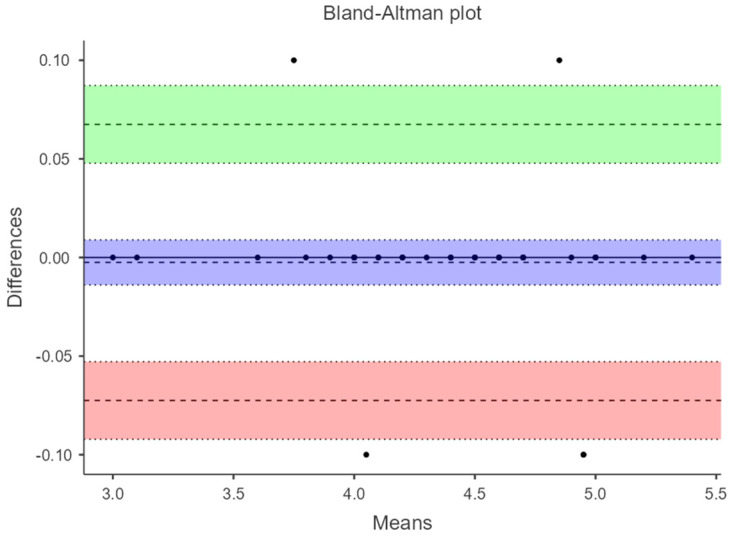
Bland–Altman plot at 6 cm from the AT to load with fixed ultrasonography vs. manual acquisition.

**Figure 4:**
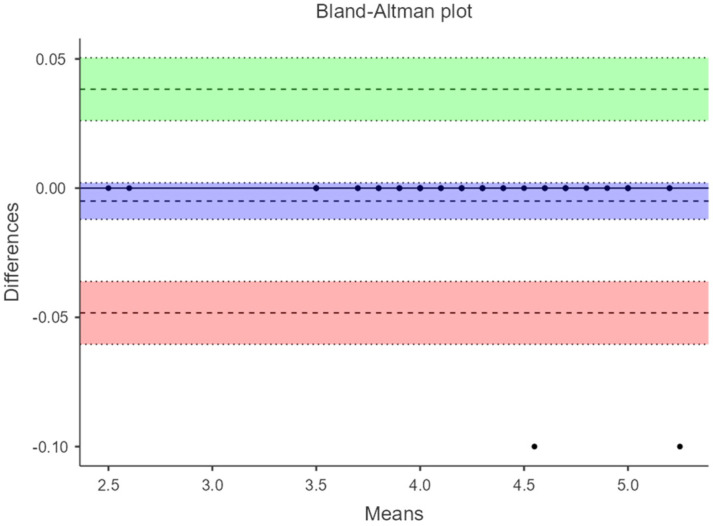
Bland–Altman plot at 4 cm from the AT to load with fixed ultrasonography vs. manual acquisition.

**Figure 5:**
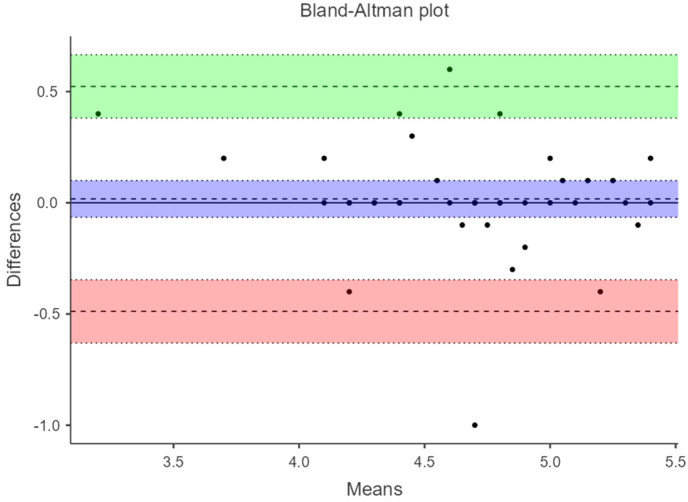
Bland–Altman plot at 6 cm from the AT to load with fixed ultrasonography examinator 1 vs. examinator 2.

Figure 3 and Figure 4 show the Bland–Altman plots where the agreement between manual acquisition and fixed ultrasound measurement under load with maximum ankle dorsiflexion is evaluated. The difference in measurements has an amplitude of −0.0025 (−0.0725 to 0.0675) and −0.005 (−0.0483 to 0.0383), respectively. Figure 5 displays the plot evaluating the agreement of fixed ultrasound measurements by two different examiners with a difference amplitude of 0.0175 (0.488 to 0.523).

## 4. Discussion

This study aimed to validate the reliability of fixed ultrasonography compared to manual acquisition in measuring the thickness of the Achilles tendon (AT) under both non-weight-bearing (patient at rest on the examination table) and weight-bearing (patient in a standing position performing maximum ankle dorsiflexion) conditions. The findings indicate that ultrasound measurements of the AT thickness, taken from healthy adult participants without any prior AT pathology, exhibit excellence, even when conducted by different evaluators and under load-bearing conditions. The results from the three figures demonstrate minimal differences between measurements and narrow ranges, implying a reasonably high level of agreement among the methods or examiners assessed in these measurements.

Our findings are consistent with previous research that reported the high reliability of ultrasonographic measurements of the Achilles tendon. For instance, van Schie et al. (2010) demonstrated the high reproducibility of ultrasound-based measurements of the tendon structure, highlighting the potential of ultrasonography in providing consistent and accurate assessments of tendon morphology [7]. Similarly, O’Connor et al. (2004) reported excellent inter-rater reliability for ultrasound assessments of tendons in asymptomatic volunteers, suggesting that ultrasound is a dependable method for evaluating tendon thickness across different examiners [26].

When comparing these findings with the literature regarding measurements at rest, Ríos-Díaz et al. (2010) found an excellent ICC for inter-rater reliability but with a wider confidence interval (ICC = 0.94; 95% CI = 0.58 to 0.98) [21], and Baño-Aledo ME et al. obtained an ICC of (ICC = 0.98; 95% CI = 0.96–0.99) [29]. An explanation for why our study obtained lower reliability could be that these authors obtained measurements in a transverse view of the AT, whereas the present study explored the tendons longitudinally. Longitudinal measurements are generally more appropriate for assessing the Achilles tendon because they allow for a more complete visualization of the tendon structure, capturing both the tendon thickness and its relationship with the bony insertion. In contrast, transverse measurements might not provide as much anatomical context and could lead to less precise assessments of tendon morphology. Longitudinal views also facilitate consistent identification of the same anatomical landmarks, which is crucial for reliability, especially in load-bearing conditions, where tendon behavior might vary. These measurements were obtained in the longitudinal section because it is the only method that allows the examiner to record the distance from the point where the thickness is measured to the bony insertion.

On the other hand, Heres et al. evaluated the lateral vastus muscle freehand and with ultrasound probe fixation, showing inconclusive reliability data, but they concluded that probe fixation enhances the field of view (FOV), and their measurements yielded significant values (*p* ≤ 0.05) [5]. Concerning our results, our findings contribute a different way of assessing the AT and enhance the clinical applicability of quantitative musculoskeletal ultrasound in settings other than the clinic and assessments beyond the examination table.

The clinical implications of these findings are significant. The accurate and reliable measurement of Achilles tendon thickness is crucial for diagnosing and managing tendinopathies. Tendinopathy, often resulting from overuse, can lead to significant morbidity if not properly assessed and treated. The use of fixed ultrasonography, which reduces operator dependency, can enhance diagnostic precision and treatment planning. For instance, clinicians can rely on these measurements to monitor tendon health over time, tailor rehabilitation programs, and make informed decisions about the need for interventions. Additionally, the ability to obtain reliable measurements under load-bearing conditions provides valuable insights into tendon function during activities. This can be particularly useful for athletes or individuals engaged in repetitive activities, as it allows for a more functional assessment of the tendon. By evaluating the tendon in a state that closely mimics daily activities, clinicians can better understand the mechanical stresses placed on the tendon and develop more effective, individualized treatment plans [30,31,32,33].

At a clinical level, sonographers encounter difficulties in obtaining good tendon images due to anatomical differences among subjects and image acquisition techniques [6,21]. The most common anatomical difficulties include the presence of a thick subcutaneous adipose layer that blurs the tendon boundary or bone morphology that might create acoustic shadowing [21,26]. On the other hand, technical difficulties that can distort images in thickness measurements include probe pressure and probe inclination [26]. It is important to be aware that ultrasound has variability depending on the operator. In a study comparing the magnetic resonance imaging of the AT, Kruse et al. demonstrated alterations in tendon morphology and that the resulting measurements depended on the amount of pressure applied to the transducer. For this reason, we consider that taking measurements with fixed ultrasonography could minimize errors.

One of the main strengths of this study is the use of a novel device to fix the ultrasound probe, ensuring consistent positioning and reducing variability. This fixed ultrasound device can have important applications in routine clinical practice. By reducing operator dependency and minimizing probe movement, clinicians can obtain more reliable measurements across different sessions, which is particularly beneficial for monitoring the progression of conditions such as tendinopathy or assessing recovery post-intervention. Furthermore, this technology could be integrated into standard protocols for evaluating musculoskeletal structures, offering a more consistent and objective method for ultrasound-based diagnostics, especially in busy clinical settings where multiple operators might be involved. This innovation addresses a common limitation in ultrasonographic assessments, where manual handling of the probe can introduce significant variability. However, there are some limitations. The sample size, while sufficient to detect significant reliability, was relatively small and homogeneous, limiting the generalizability of the findings. Future studies should include larger and more diverse populations to validate these findings across different demographic groups and clinical conditions. Additionally, the study did not assess the reliability of the method in pathological tendons, which should be addressed in future research to confirm the applicability of fixed ultrasonography in clinical settings involving tendinopathy or other tendon disorders.

The main bias in this study was the operator probe pressure in measurements without a device between subjects, as it could not be quantified. The measurements were only taken in longitudinal sections as it was the only quantitative way to measure the thickness at the correct distance from the calcaneal insertion without losing the cutting point.

A limitation of the present study is that the sample consisted exclusively of young university students. This may not adequately represent the general population, especially individuals of older age or with different levels of physical activity. Future studies should include a larger and more diverse sample to evaluate the reliability of ultrasound measurements of the Achilles tendon in different age groups and clinical conditions.

This study focused on evaluating reliability in healthy individuals. To determine the clinical relevance of these measurements, it is crucial to conduct comparative studies in individuals with tendinopathies or other Achilles tendon pathologies. This would allow for the assessment to determine whether the reliability of measurements is maintained in the presence of pathological conditions and if the observed differences can influence clinical diagnosis and treatment.

## 5. Conclusions

In conclusion, fixed ultrasonography provides highly reliable measurements of Achilles tendon thickness under both non-weight-bearing and weight-bearing conditions. This method offers a valuable tool for clinical practice, enhancing the accuracy of tendon assessments and potentially improving patient care. The introduction of a fixed ultrasound device addresses a significant limitation in current ultrasonographic practices by reducing operator variability, thus contributing to more consistent and reliable tendon evaluations. Further research is warranted to explore its full potential in diverse clinical populations and its long-term impact on clinical outcomes.

## Figures and Tables

**Table 1:** Characteristic of population.

Gender (n)	Age (y) (Mean/SD)	Weight (k) (Mean/SD)	Height (cm) (Mean/SD)	BMI (Mean/SD)
Male (6)	22.5 ± 3.12	79.8 ± 13.91	167 ±6.79	25.1 ± 2.99
Female (14)	22.6 ± 1.95	63.8 ± 6.94	178 ± 6.16	23 ± 2.77
Total (20)	22.55 ± 2.32	68.6 ± 12.51	170 ± 8.07	23.61 ± 2.97

n (number), y (years), SD (standard deviation), k (kilogram), cm (centimeter), BMI (body mass index).

**Table 2:** Descriptive variable Achilles thickness with manual acquisition and with fixed ultrasonography.

		Intra-Rater				Inter-Rater		
Variables	US Acquisition	Examiner 1	Examiner 2			Mean (SD) 95% CI	α Cronbach	* Mean Difference 95% LOA
		Mean (SD) 95% CI	Mean (SD) 95% CI	ICC_(1-1)_ (95% CI)	*p*-Value			
4 cm At rest	manual acquisition	4.50 (0.54)	4.49 (0.60)	0.91 (0.86–0.95)	<0.001	4.51 (0.528)	0.998	0.005 (−0.104 to 0.094)
6 cm At rest	manual acquisition	4.63 (0.455)	4.66 (0.611)	0.895 (0.861–0.945)	<0.001	4.63 (0.450)	0.996	0.02 (−0.0905 to 0.13)
4 cm At rest	fixed ultrasonography	4.58 (0.556)	4.66 (0.579)	0.926 (0.882–0.957)	<0.001	4.58 (0.543)	0.997	0.000 (−0.109 to 0.109)
6 cm At rest	fixed ultrasonography	4.67 (0.461)	4.66 (0.510)	0.909 (0.861–0.945)	<0.001	4.67 (0.460)	0.997	0.01 (−0.0872 to 0.1072)
4 cm At load/DF	fixed ultrasonography	4.24 (0.624)	4.36 (0.614)	0.894 (0.826–0.94)	<0.001	4.24 (0.626)	1.000	−0.005 (−0.0483 to 0.0383)
6 cm At load/DF	fixed ultrasonography	4.39 (0.531)	4.36 (0.631)	0.876 (0.802–0.928)	<0.001	4.39 (0.533)	0.999	−0.0025 (−0.0725 to 0.0675)

Variables: Achilles tendon thickness in centimeters (cm). SD: standard deviation. 95% CI: 95% confidence Interval; * Mean differences in millimeters (mm); LOA: Limit of agreement. DF: measurement was taken in a standing position with maximum ankle dorsiflexion.

## Data Availability

The data presented in this study are available on request from the corresponding author due to it being part of an unpublished thesis currently under embargo.
